# A 4-Phenoxyphenol Derivative Exerts Inhibitory Effects on Human Hepatocellular Carcinoma Cells through Regulating Autophagy and Apoptosis Accompanied by Downregulating α-Tubulin Expression

**DOI:** 10.3390/molecules22050854

**Published:** 2017-05-21

**Authors:** Wen-Tsan Chang, Wangta Liu, Yi-Han Chiu, Bing-Hung Chen, Shih-Chang Chuang, Yen-Chun Chen, Yun-Tzh Hsu, Mei-Jei Lu, Shean-Jaw Chiou, Chon-Kit Chou, Chien-Chih Chiu

**Affiliations:** 1 Division of General and Digestive Surgery, Department of Surgery, Kaohsiung Medical University Hospital, Kaohsiung 807, Taiwan; wtchang@kmu.edu.tw (W.-T.C.); chuangsc@cc.kmu.edu.tw (S.-C.C.); 2 Department of Surgery, School of Medicine, College of Medicine, Kaohsiung Medical University, Kaohsiung 807, Taiwan; 3 Department of Biotechnology, Kaohsiung Medical University, Kaohsiung 807, Taiwan; liuwangta@kmu.edu.tw (W.L.); bhchen@kmu.edu.tw (B.-H.C.); shiny_0224@yahoo.com.tw (Y.-C.C.); 4a1h0007@stust.edu.tw (Y.-T.H.); fjm11037@yahoo.com.tw (M.-J.L.); fatchou1988@hotmail.com (C.-K.C.); 4 Department of Nursing, St. Mary’s Junior College of Medicine, Nursing and Management, Yi-Lan 266, Taiwan; chiuyiham@smc.edu.tw; 5 The Institute of Biomedical Sciences, National Sun Yat-Sen University, Kaohsiung 804, Taiwan; 6 Faculty of Medicine, College of Medicine, Kaohsiung Medical University, Kaohsiung 807, Taiwan; 7 Transplantation Center, Kaohsiung Medical University Hospital, Kaohsiung 807, Taiwan; 8 Department of Biochemistry, College of Medicine, Kaohsiung Medical University, Kaohsiung 807, Taiwan; sheanjaw@cc.kmu.edu.tw; 9 Translational Research Center, Cancer Center, Department of Medical Research, Kaohsiung Medical University Hospital, Kaohsiung Medical University, Kaohsiung 807, Taiwan; 10 Research Center for Environment Medicine, Kaohsiung Medical University, Kaohsiung 807, Taiwan; 11 Graduate Institute of Medicine, College of Medicine, Kaohsiung Medical University, Kaohsiung 807, Taiwan; 12 Department of Biological Sciences, National Sun Yat-sen University, Kaohsiung 804, Taiwan

**Keywords:** apoptosis, autophagy, ERK, 4-phenoxyphenol, HCC, α-tubulin, microtubule

## Abstract

Hepatocellular carcinoma (HCC) is a leading cancer worldwide. Advanced HCCs are usually resistant to anticancer drugs, causing unsatisfactory chemotherapy outcomes. In this study, we showed that a 4-phenoxyphenol derivative, 4-[4-(4-hydroxyphenoxy)phenoxy]phenol (4-HPPP), exerts an inhibitory activity against two HCC cell lines, Huh7 and Ha22T. We further investigated the anti-HCC activities of 4-HPPP, including anti-proliferation and induction of apoptosis. Our results showed that higher dosage of 4-HPPP downregulates the expression of α-tubulin and causes nuclear enlargement in both the Huh-7 and Ha22T cell lines. Interestingly, the colony formation results showed a discrepancy in the inhibitory effect of 4-HPPP on HCC and rat liver epithelial Clone 9 cells, suggesting the selective cytotoxicity of 4-HPPP toward HCC cells. Furthermore, the cell proliferation and apoptosis assay results illustrated the differences between the two HCC cell lines. The results of cellular proliferation assays, including trypan blue exclusion and colony formation, revealed that 4-HPPP inhibits the growth of Huh7 cells, but exerts less cytotoxicity in Ha22T cells. Furthermore, the annexin V assay performed for detecting the apoptosis showed similar results. Western blotting results showed 4-HPPP caused the increase of pro-apoptotic factors including cleaved caspase-3, Bid and Bax in HCC cells, especially in Huh-7. Furthermore, an increase of autophagy-associated protein microtubule-associated protein-1 light chain-3B (LC3B)-II and the decrease of Beclin-1 and p62/SQSTM1 were observed following 4-HPPP treatment. Additionally, the level of γH2A histone family, member X (γH_2_AX), an endogenous DNA damage biomarker, was dramatically increased in Huh7 cells after 4-HPPP treatment, suggesting the involvement of DNA damage pathway in 4-HPPP-induced apoptosis. On the contrary, the western blotting results showed that treatment up-regulates pro-survival proteins, including the phosphorylation of protein kinase B (Akt) and the level of survivin on Ha22T cells, which may confer a resistance toward 4-HPPP. Notably, the blockade of extracellular signal-regulated kinases (ERK), but not Akt, enhanced the cytotoxicity of 4-HPPP against Ha22T cells, indicating the pro-survival role of ERK in 4-HPPP-induced anti-HCC effect. Our present work suggests that selective anti-HCC activity of 4-HPPP acts through induction of DNA damage. Accordingly, the combination of ERK inhibitor may significantly enhance the anti-cancer effect of 4-HPPP for those HCC cells which overexpress ERK in the future.

## 1. Introduction

Hepatocellular carcinoma (HCC) is one of the most common causes of cancer death around the world, particularly in Asia. In Taiwan, HCC is responsible for about 7000 cancer-associated deaths, and some 8000 new cases are diagnosed annually [1]. Regardless of disease stages, partial surgical resection, percutaneous ablation, and systemic chemotherapeutic administration remain important parts of the treatment paradigm for liver cancers. However, limitations to their use exist, such as maximum lifetime dose, tumor resistance, allergic reactions and serious complications. Furthermore, the acquired chemoresistance in an advanced stage of HCC frequently determines poor survival rate of HCC patients [2]. To meet these challenges, developing effective chemotherapeutic treatments against HCC is desirable and warranted.

The cell division cycle is composed of a series of events that take place in a cell leading to its division and duplication. The process of mitosis, including nuclear division and cytokinesis, is complex and highly regulated. Microtubules, composed of α/β-tubulin heterodimers, are one of the essential components of the cytoskeleton that play a key role in various cellular processes such as development and maintenance of cell shape, intracellular transport, cell signaling, and mitosis [3]. Due to their important roles in a variety of cellular functions, microtubules become one of the most important targets for anti-cancer therapy [4]. Recently, Gasparotto et al. identified the phenolic analog 3-cyclopropylmethyl-7-phenyl-3*H*-pyrrolo[3,2-*f*]quinolin-9(6*H*)-one (MG-2477), as a potent cytotoxic anti-microtubule agent that might affect the survival of human tumor cell lines with interfering microtubules [5]. Viola et al. also reported that MG-2477 is highly effective in inducing autophagy of A549 non-small cell lung carcinoma cells through inhibition of the Akt/mTOR pathway and delayed apoptosis [6].

Phenolic compounds are ubiquitous to plant material and have utility as pharmaceutical drugs [7,8,9]. Phenolic compounds have been reported to modulate key proteins in the signaling cascades related to differentiation, proliferations, metastasis and apoptosis for the treatment of cancer [10,11]. For example, camptothecin (CPT), isolated from the plant *Camptotheca acuminata*, has been shown to exhibit anti-cancer activities [12]. The two CPT derivatives, irinotecan and topotecan, which have hydrophilic groups, are widely used as clinical anti-cancer drugs [13,14]. Another phenolic derivative, 9-bis[2-(pyrrolidin-1-yl)ethoxy]-6-{4-[2-(pyrrolidin-1-yl)ethoxy]phenyl}-11*H*-indeno[1,2-c]quinolin-11-one (BPIQ), has been shown to exert inhibitory potential against several common cancer cells, including non-small lung cancer cells (NSCLC) [15], hepatocellular carcinoma (HCC) [16] and retinoblastoma (Rb) [17] tumor cells using a 3-(4,5-dimethylthiazol-2-yl)-2,5-diphenyl-tetrazolium bromide (MTT)-based proliferation assay. Among phenolic compounds, phenoxyphenol derivatives are reported to possess the capacity to prevent and treat colon and prostate cancers [18,19]. Specifically, Parsai et al. revealed that 4-phenoxyphenol derivatives could bind to the active site of matrix metalloproteinases (MMP)-9 and cyclooxygenase (COX)-2 with a high affinity, thus possessing anti-metastasis and anti-inflammatory properties [20]. The underlying anti-cancer mechanism of phenoxyphenol derivatives remains far from clear. In this report, we sought to evaluate the inhibitory effects of phenoxyphenol analog, 4-[4-(4-hydroxyphenoxy)phenoxy]phenol (4-HPPP), on the HCC cell lines Ha22T and Huh7 cells. We also investigated the mechanism of 4-HPPP-induced inhibition of HCC cells, including the regulation of α-tubulin, apoptosis, autophagy and survival related proteins, such as Akt, ERK, and survivin. Finally, we summarize that 4-HPPP may be a promising agent capable of inhibiting proliferation of HCC cells, suppressing α-tubulin expression, promoting nuclei enlargement of HCC cells, inducing apoptosis and formation of autolysosomes, activating expression of γH2AX and down-regulating the pro-survival pathways.

## 2. Results

### 2.1. The Anti-Proliferative Effects of 4-HPPP on HCC Cells

**Figure 1:**
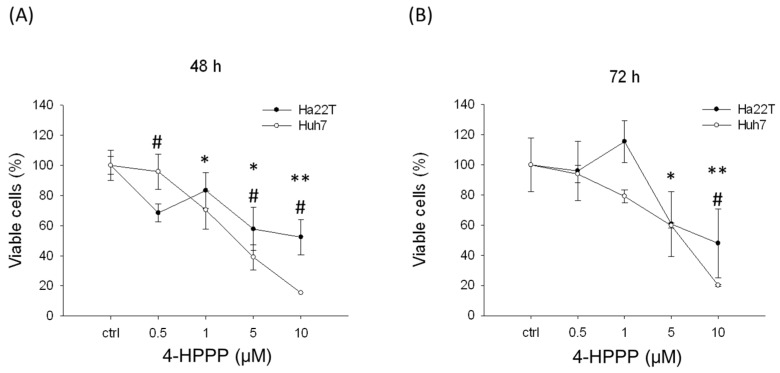
The inhibitory effect of 4-HPPP on the short-term proliferation of HCC cells. Two HCC cells. Huh7 and Ha22T were seeded on a 12-well plate and treated with indicated concentrations (from 0.5 to 10 μM) of 4-HPPP for 48 h (**A**) and 72 h (**B**) respectively. Ctrl: Vehicle as a control group. * *p* < 0.05 and ** *p* < 0.001 for Huh-7; # *p* < 0.05 for Ha22T.

The anti-proliferative effect of 4-HPPP on hepatocellular carcinoma cell lines, namely Huh7 and Ha22T, was first evaluated using a trypan blue exclusion assay. The proliferation rates of Huh7 cells at 48 h were: vehicle control, 100 ± 10.10%; 0.5 μM of 4-HPPP, 95.86 ± 11.72%; 1 μM of 4-HPPP, 70.41 ± 12.55%; 5 μM of 4-HPPP, 39.05 ± 8.37%; and 10 μM of 4-HPPP, 15.38 ± 0.00%. The proliferation rate of Huh7 cells at 72 h was: vehicle control, 100.00 ± 17.75%; 0.5 μM of 4-HPPP, 93.92 ± 5.92%; 1 μM of 4-HPPP, 79.09 ± 4.30%; 5 μM of 4-HPPP, 59.70 ± 1.61%; and 10 μM of 4-HPPP, 20.15 ± 0.54%. However, the proliferation rates of Ha22T cells at 48 h for vehicle control, 0.5, 1, 5 and 10 μM of 4-HPPP were 100.00 ± 10.10%, 68.45 ± 5.89%, 83.33 ± 11.79%, 57.74 ± 14.31% 52.38 ± 11.79%, respectively. And those at 72 h for vehicle control, 0.5, 1, 5 and 10 μM of 4-HPPP were 100.00 ± 0%, 95.96 ± 19.70%, 115.48 ± 14.01%, 60.73 ± 21.64%, 47.95 ± 22.77%, respectively (Figure 1A,B).

**Figure 2:**
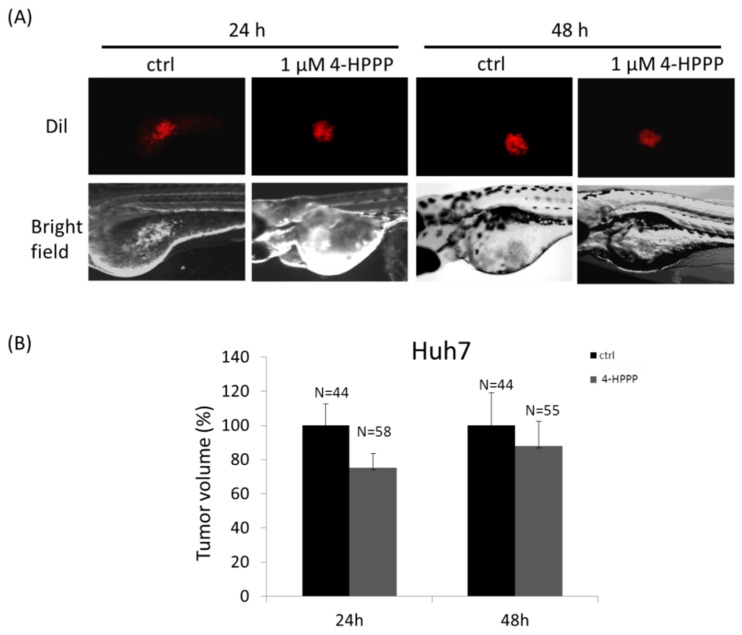
The inhibitory effect of 4-HPPP on anti-HCC using in vivo zebrafish xenograft assay. (**A**) A total of 200 Huh7 cells was microinjected into the yolk sac of the zebrafish embryos at 2 dpf (days post fertilization) and exposed to 1 μM of 4-HPPP for 24 and 48 h respectively. (**B**) The quantitative analysis of tumor volume of (**A**). *N* stands for sample size.

The half-maximum inhibitory concentration (IC_50_) values were found to be 3.61 and 6.22 μM in Huh7 cells at 48 and 72 h and 9.18 μM for Ha22T cells at 72 h. Our results indicated that 4-HPPP reduced the proliferation of both cells in vitro in a concentration-dependent manner. Additionally, these hepatocellular carcinoma cell lines had discrepant sensitivities to 4-HPPP. The in vivo zebrafish-based tumor xenograft was also conducted. The inhibitory effect of 4-HPPP on zebrafish-based xenograft was moderate, and there is no statistically significant difference between control and 4-HPPP treatment (*p* > 0.05) (Figure 2).

### 2.2. The Assessment of 4-HPPP-Induced Long-Term Anti-Proliferation of HCC 

**Figure 3:**
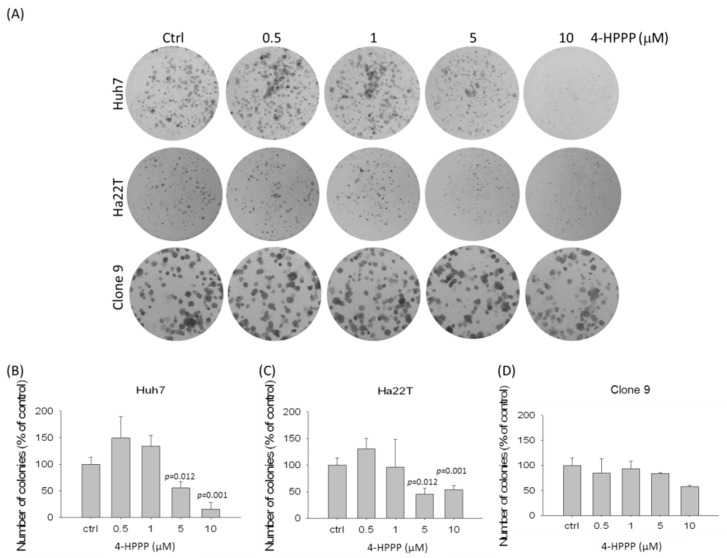
The inhibitory effect of 4-HPPP on the long-term proliferation of human HCC and rat hepatocyte cells. HCC cell lines Huh7 and Ha22T, and the rat hepatocyte Clone 9 were treated with indicated concentrations (from 0.5 to 10 μM) of 4-HPPP for 7 and 10 days respectively. Afterward, cells were fixed with 4% paraformaldehyde and stained with Giemsa dye. (**A**) The representative results of colony formation of Huh7, Ha22T and Clone 9 cells following 4-HPPP treatment. (**B**–**D**) The quantitative analysis of (**A**). Data were statistically analyzed with the Student t-test. *p* value, vehicle control vs. 4-HPPP treatments. Ctrl indicates the vehicle control.

We conducted a colony formation assay to examine the effect of 4-HPPP on the long-term proliferation of HCC cells. As shown in Figure 3, the results revealed that colony numbers of two HCC cell lines, Huh7 and Ha22T, were dramatically decreased in the presence of the indicated concentrations (from 0.5 to 10 μM) of 4-HPPP, suggesting the persistently inhibitory potential of 4-HPPP against HCC cells. Interestingly, the rat hepatocyte Clone 9 cells were less sensitive to the 4-HPPP treatment compared to Huh7 cells, suggesting the selective anti-proliferative effect of 4-HPPP (Figure 3).

### 2.3. 4-HPPP Inhibits α-Tubulin Expression

**Figure 4:**
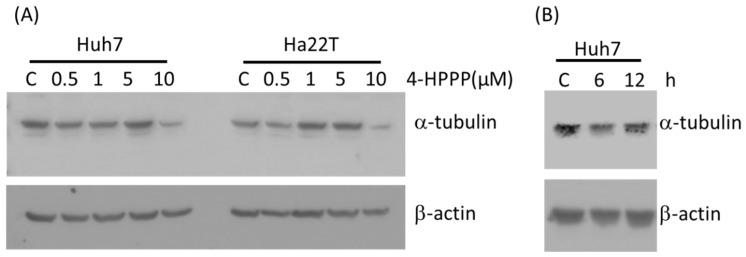
The effect of 4-HPPP on tubulin expression of HCC cells. (**A**) Expression of α-tubulin protein in HCC cells Huh7 and Ha22T cells treated with indicated concentrations of 4-HPPP. (**B**) Expression of α-tubulin in Huh7 cells treated with 10 μM of 4-HPPP for 6 and 12 h respectively. The results of Western blot analyses of α-tubulin were from representative samples. β-actin as an internal control. C stands for vehicle control.

To evaluate if 4-HPPP interfered with the microtubule network, we first examined its effects on cultured cells by western blotting assay. Following 24 h of treatment with 0.5 to 10 μM of 4-HPPP, expression levels of α-tubulin were decreased on Huh7 and Ha22T cells when treated with the highest concentration (Figure 4A). Furthermore, the time course assay showed that the protein level of α-tubulin was decreased at 6 h of 10 μM 4-HPPP administration in Huh7 cells (Figure 4B).

### 2.4. 4-HPPP Induces Nuclei Enlargement of HCC Cells

**Figure 5:**
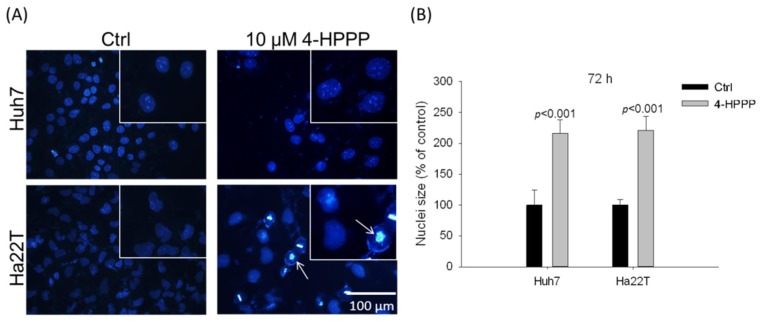
4-HPPP induces nuclei enlargement of HCC cells. Two HCC cell lines Huh7 and Ha22T were treated with 10 μM of 4-HPPP for 72 h respectively. Afterward, cells were stained with 0.2 μg/mL DAPI. (**A**) The representative results of Huh7 and Ha22T cells following 4-HPPP treatment. (**B**) The quantitative analysis of nuclei size of (**A**). White arrows indicate the condensed chromatins. Magnification: 200×. *p* < 0.05 control vs. 4-HPPP was considered statistically significant.

As shown in Figure 5, the results revealed that, in the presence of 10 μM 4-HPPP, it caused a significant nuclei enlargement of HCC cells.

The results of DAPI staining showed that vehicle control and 10 μM 4-HPPP induced the percentage of nuclei enlargement of Huh7 and Ha22T cells were 100 ± 24.27%, 216.39 ± 22.41% and 100.00 ± 9.12%, 221.23 ± 21.77% at 72 h, respectively.

### 2.5. 4-HPPP Induces Apoptosis of HCC Cells

**Figure 6:**
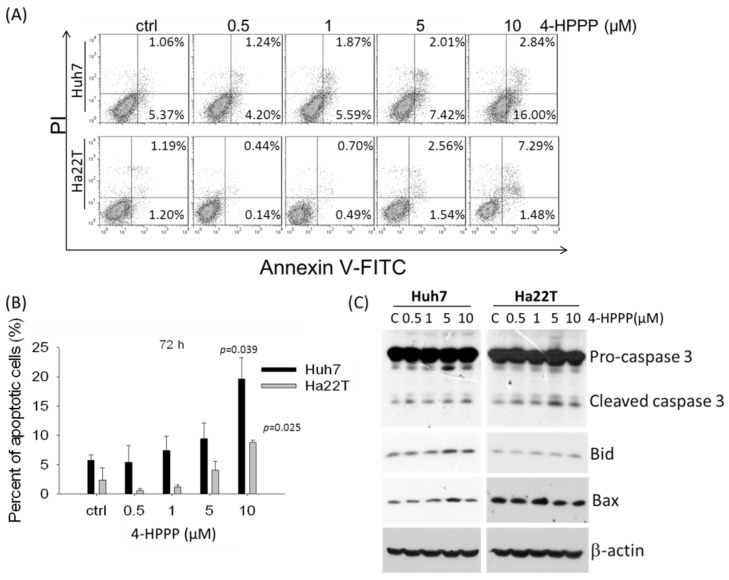
The assessment of 4-HPPP-induced apoptosis in HCC cells. Flow cytometry-based annexin V staining for detecting apoptosis. (**A**) Cells were treated with indicated concentrations of 4-HPPP for 72 h respectively. (**B**) The quantitative analysis of apoptotic cells of (**A**). *p* < 0.05 control vs. 4-HPPP was considered statistically significant. (**C**) The changes of pro-apoptotic factors including cleaved caspase-3, Bid and Bax in HCC cell lines following 4-HPPP treatment. β-actin as an internal control for ensuring equal loading.

The previous study showed that 4-HPPP inhibits proliferation of HCC cells, so we further examined whether 4-HPPP-induced growth inhibition of Huh7 and Ha22T cells was mediated by apoptosis. Huh7 and Ha22T cells were treated with indicated concentrations of 4-HPPP for 72 h. Apoptotic cells with the externalization of phosphatidylserine (PS) were detected by Annexin V staining following treatment with 0, 0.5, 1, 5 and 10 µM of 4-HPPP. The dual fluorescence Annexin V conjugated with FITC and PI indicated non-apoptotic or necrotic cells (Annexin V^−^/PI^+^), healthy cells (Annexin V^−^/PI^−^), the early stage of apoptotic cells (Annexin V^+^/PI^−^), and the late stage of apoptotic cells (annexin V^+^/PI^+^). The percentages of early-/late- apoptotic cells were 5.74 ± 0.91%, 5.44 ± 2.83%, 7.46 ± 2.40%, 9.43 ± 2.73% and 19.64 ± 3.63% on Huh7 cells; and 2.39 ± 2.13%, 0.58 ± 0.40%, 1.18 ± 0.36%, 4.11 ± 1.50% and 8.77 ± 0.40% of Ha22T cells respectively (Figure 6). The above results showed 4-HPPP treatments elicited a significant induction of apoptosis on Huh7 and Ha22T cells.

### 2.6. 4-HPPP-Induced Formation of Autolysosomes and the Expression of Autophagy Marker Protein in HCC Cells

**Figure 7:**
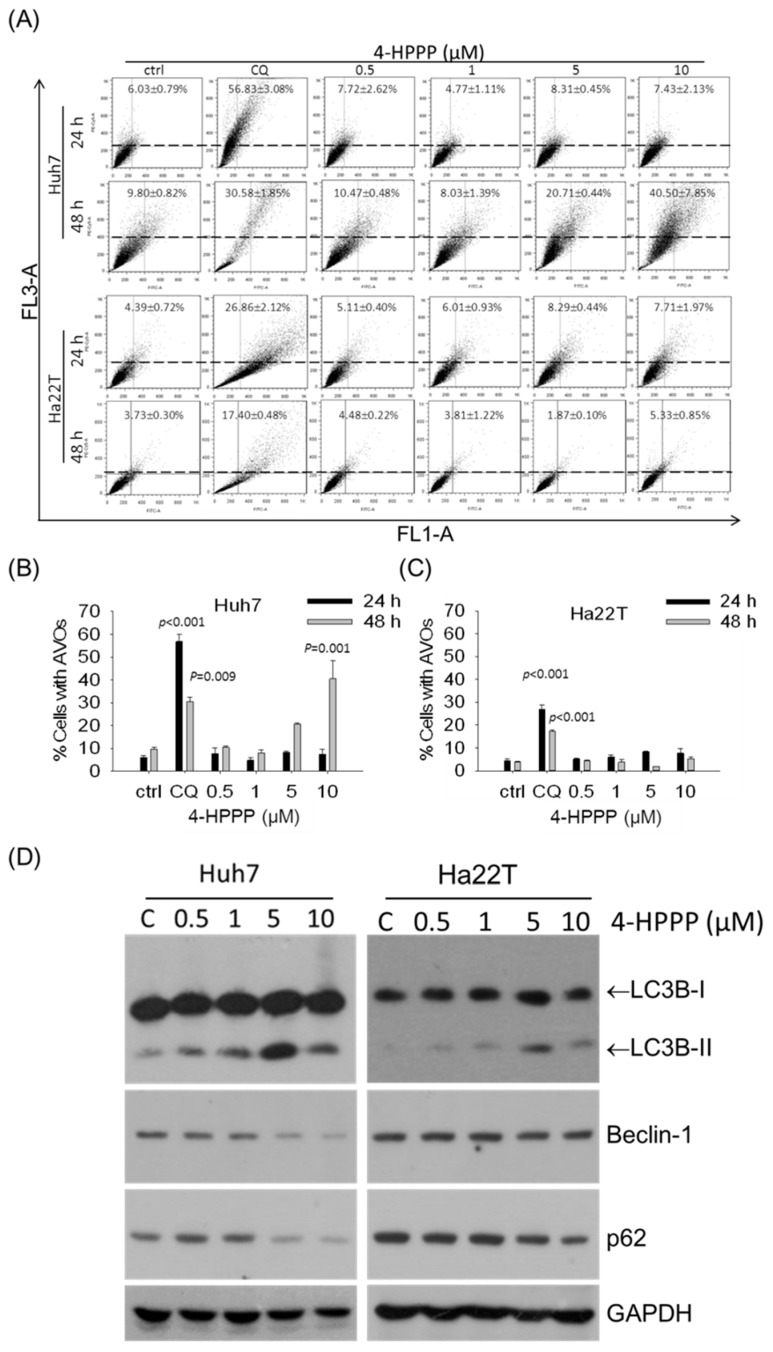
Autophagy assessment in 4-HPPP-treated HCC cells. (**A**) Huh7 and Ha22T cells were treated with 4-HPPP for 24 and 48 h respectively, and then subject to the autophagy assessment of flow cytometry-based AO staining. The formation of acidic vesicular organelles (AVOs) analyzed using a flow cytometry. (**B**,**C**) The quantitative analysis of AVOs of (**A**). 100 μM of chloroquine (CQ) as a positive control. (**D**) The western blot assay showed the expression changes of autophagy-associated protein Beclin-1, p62, LC3B-I and its cleaved form LC3B-II following treatment with indicated concentrations of 4-HPPP for 48 h. GAPDH was used as an internal control for ensuring equal loading.

To understand whether autophagy is induced by 4-HPPP, we examined the formation of acidic vesicular organelles (AVOs, which include autolysosomes) and the autophagic marker phosphatidylethanolamine- microtubule-associated protein-1 light chain-3B-II (LC3B-II) in Huh7 and Ha22T cells in the presence of 0–10 μM of 4-HPPP. As shown in Figure 7A–C, the percentage of AVO-positive Huh7 cells significantly increased in the presence of 10 μM of 4-HPPP after 48 h. Figure 7D demonstrates that the western blotting analysis results were consistent with those from the AVOs detections. The LC3B-II involved in the autophagic process exhibited a higher expression level in Huh7 cell line in the presence of 0–10 μM of 4-HPPP.

### 2.7. 4-HPPP Induces the Activation of γH2AX and Down-Regulates the Pro-Survival Pathways

**Figure 8:**
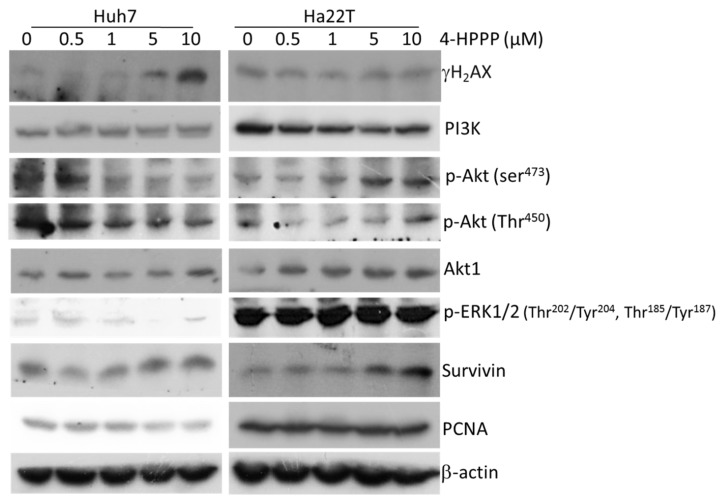
The changes of the survival-related proteins and DNA damage in 4-HPPP-treated HCC cells. Huh7 and Ha22T cells were seeded and treated with indicated concentrations of 4-HPPP for 24 h respectively. Afterward, cells were harvested and lysed. The protein lysates were resolved and analyzed by 10% and 12% sodium dodecyl sulfate-polyacrylamide gel electrophoresis (SDS-PAGE) and Western blot assay respectively. β-actin as an internal control for equal loading.

To gain insight into the mechanism underlying the cytotoxic effect of 4-HPPP on Huh7 and Ha22T cells, we next examined the changes in signal transduction pathways plausibly involved in mediating 4-HPPP action. The protein levels of γH2AX, PI3K, p-Akt, Akt, p-ERK, Survivin and proliferating cell nuclear antigen (PCNA) were determined by western blotting. As shown in Figure 8, the increase of DNA damage marker γH2AX was observed following 4-HPPP treatment in Huh7 cells, but not Ha22T cells. Also shown in Figure 8, 4-HPPP caused a decrease of pro-survival factors survivin and PCNA, and the threonine/serine kinase p-Akt (Ser^473^ and Thr^450^) in Huh7 cells. In contrast, a significant trend of induction on p-Akt (Ser^473^ but not Thr^450^) and survivin levels were observed in Ha22T cells. Furthermore, the significantly high level of p-ERK in both control and 4-HPPP-treated Ha22T cells were observed, suggesting that it could be the reason that Ha22T exhibited less sensitivity to 4-HPPP treatments.

### 2.8. ERK Blockade Rescues 4-HPPP-Inhibition of Ha22T Cells

**Figure 9:**
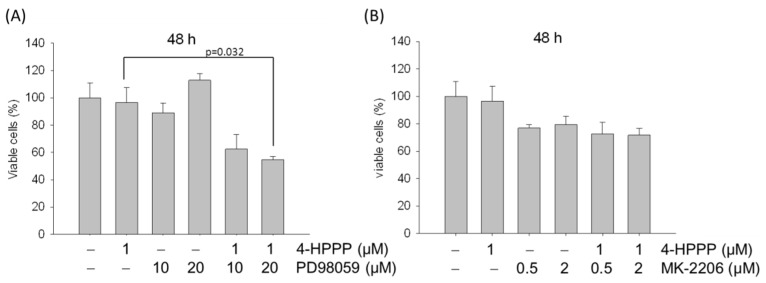
The effect of MEK1/ERK and Akt inhibitors on 4-HPPP-induced inhibition of Ha22T cells. Ha22T cells were subject to treatment with 4-HPPP alone or ERK and Akt inhibitors for 6 h prior to 4-HPPP administration for 48 h. The result of trypan blue dye assay is represented. Specific inhibitors, PD98059 for MEK1/ERK and MK-2206 for Akt, before 4-HPPP administration respectively.

The previous results showed that Ha22T cells display a less-sensitive response to 4-HPPP treatments. We further examined if activation of ERK or Akt was directly involved in 4-HPPP-induced cytotoxicity of HCCs. To this end, we studied the effect of pharmacologic inhibitors for mitogen-activated protein or extracellular signal-regulated kinase kinase 1 (MEK1)/ERK (PD98059) and Akt (MK-2206) on 4-HPPP-induced cell death. As shown in Figure 9A, the percent viable cells were decreased from 96.58 ± 10.88% to 54.70 ± 2.42% (1 μM 4-HPPP alone vs. 4-HPPP with 20 μM PD98059 respectively). Therefore, the pre-treatment of MEK1/ERK inhibitor PD98059 significantly enhanced the 4-HPPP-induced cytotoxicity to Ha22T cells. However, the pre-treatment with pan-Akt inhibitor did not significantly affect the cell viability as compared with 4-HPPP treatment alone (Figure 9B). Our study implies that treatment with 4-HPPP and PD98059 synergistically enhances Ha22T cell death, suggesting that targeting ERK pathways, but not the Akt pathway, may further improve the efficacy of 4-HPPP treatment in the 4-HPPP-insensitive Ha22T cell.

## 3. Discussion

HCC patients are usually diagnosed at advanced stages in Taiwan [1]. Current chemotherapeutic treatments often cause resistance in HCC cells, causing poor prognosis and low survival rates [21]. Therefore, the screening of anti-HCC compounds without significant resistance effects is urgent.

Phenolic compounds have been shown to exert anti-cancer activities. For example, the two well-known phenolic antibiotics, clioquinol (iodochlorhydroxyquin) and nitroxoline (8-hydroxyl-5-nitroquinoline), were reported to exhibit both anti-proliferative and anti-migratory activities against cholangiocarcinoma cells [22]. Previously, Tseng’s work examined the anti-cancer effects of a panel of synthetic phenolic derivatives against six human common cancer cell lines, including hepatocellular carcinoma SKHep using a MTT-based proliferation assay; that indicated synthetic phenolic derivatives exert a significant anti-HCC activity [16]. Although many phenolic compounds have been intensely studied for their antitumor effects for years, the underlying mechanism of phenolic compound-induced anti-HCC effect and the long-term inhibitory effect of the phenolic compounds on HCC are still unknown. Recently, a synthetic phenoxyphenol, 4-[4-(4-hydroxy-phenoxy)phenoxy]phenol (4-HPPP) was demonstrated in our laboratory to exert anti-cancer activity.

Little has been discussed regarding the bioactivity of phenoxyphenol compounds. Yu et al. synthesized the phenophxyphenol derivative 4-(4-phenoxyphenoxy)phenol in 1994 [23]. Since then, the bioactivities of phenoxyphenol derivatives remains unclear. In the phenoxyphenol 4-HPPP used in our study, an additional hydroxyl group is present compared to the structure of 4-(4-phenoxy-phenol)phenol synthesized by Yu et al. [23].

Recent reports have demonstrated the critical role of microtubules in regulating the segregation of chromosome during cellular division, intracellular transport, cell motility, and the maintenance of cell shape [23]. Microtubule targeting agents disrupt the tubulin dynamics by binding to distinct sites on protein tubulin, an α/β dimer that forms the core of the microtubule [3]. Many anti-cancer drugs inhibit the proliferation of cancer cells through affecting the polymerization dynamics of tubulins [6,24]. A previous study found that a certain phenolic analog, *N*-benzyl-2-[5-[4-(2-morpholin-4-ylethoxy)phenyl]pyridin-2-yl]acetamide) (KX2-391) [25] was a dual Src kinase and tubulin polymerization inhibitor and achieved modest curative effects in men with chemotherapy-naïve bone-metastatic castration-resistant prostate cancer (CRPC). In this study, we observed a moderately decreased expression of α-tubulin in both HCC cell lines following 4-HPPP treatment (Figure 4) with induced nucleus enlargement (Figure 5). As a phenoxyphenol derivative, 4-HPPP may have therapeutic potential in cancer treatment, and whether 4-HPPP interferes with microtubules witll be examined using a cellular tubulin polymerization assay in our further study.

To investigate the effect of 4-HPPP on proliferation, apoptosis, autophagy and DNA damage of HCC, two HCC cell lines, Ha22T and Huh7, were used. In the study, 4-HPPP inhibited proliferation and significantly reduced the colony number in Huh7, whereas it exerted relatively modest cytotoxicity against Ha22T cell lines, as shown by trypan blue exclusion and colony formation assay (Figure 1 and Figure 3). Interestingly, the cell growth assessment showed that 5 μM of 4-HPPP exerted a moderately inhibitory effect on cell growth of Ha22T, but it did not reach statistical difference (*p* = 0.064, *p* value > 0.05), indicating that Ha22T cells were less sensitive toward 4-HPPP than Huh7 cells.

According to results of viability assay (Figure 1) and Annexin V-staining (Figure 6), we noted that both HCC cell lines were sensitive to a low dose 0.5 μM of 4-HPPP; however, a drug resistance of Ha22T was observed at the dose of 1 μM compared to Huh7 cells. Likewise, a previous study reported that the drug-resistant cancer cells seem to be sensitive toward low doses of chemotherapy, but exert an increased resistance to higher doses of chemotherapy. For example, Caski, an epidermoid cervical carcinoma cell, showed a similar cytotoxicity to low concentrations of cisplatin (IC_30_) compared to other tested cancer cells, but exerted an increased resistance at higher concentrations [26]. Accordingly, our results suggested that the increased dose of 4-HPPP may trigger a chemoresistance in Ha22T cells.

On the other hand, the previous study also showed that microtubule inhibitors might cause the fusion failure of autophagosome and lysosome, resulting in the accumulation of LC3II [27]. In the study, we found accumulation of acidic vesicular organelles (AVOs) in 4-HPPP-treated Huh7 cells but not in Ha22T. The autophagy-related protein LC3BII and the formation of AVOs were accumulated concomitantly, suggesting that 4-HPPP enhances autophagy in Huh-7 cells.

The stages of autophagy include initiation, elongation, maturation, fusion with the lysosome, and the final degradation of damaged organelles or other macromolecules [28]. Besides the cleavage of LC3B-I (LC3B-II), an autophagosome marker, we also checked two important markers of autophagy Beclin and p62/SQSTM1 in HCC cells following 4-HPPP treatment. Beclin is a marker of the stage vesicle nuclear of autophagy process. When p62/SQSTM1 and damaged organelles are conjugated and enter into the autophagosome, the autophagy process goes further after the fusion of autophagosome and lysosome, resulting in the formation of autolysosome [29]. Finally, proteins or damaged organelles are degraded in the autolysosome [29,30]. In the study, the inconsistent accumulation of AVO and the cleavage of LC3B indicated that the autophagy was induced by 4-HPPP in both HCC cell lines, especially Huh7 cells. However, the fusion of lysosome and autophagosome seems not to be initiated in Ha22T cells. In contrast, the significant decrease of P62 and Beclin was detected in Huh7 cells, suggesting that 4-HPPP may facilitate the autophagy process in Huh7 but not in Ha22T.

Anti-cancer drugs inhibit cancer cells through the induction of apoptosis or autophagic death. However, recent evidence has suggested both types of cell death in cancer cells could be induced concomitantly. For example, Zhang’s work suggested that arenobufagin (a bufadienolide from venom secreted by the Argentine toad *Bufo arenarum*) inhibited HepG2 cells, and multidrug resistant HepG2/AMD cells involved the cross-talk between apoptosis and autophagy through inhibiting the PI3K/Akt/mTOR pathway [31]. On the contrary, Wang’s work showed that berberine (a small molecule derived from the Chinese herb *Coptidis rhizoma*) induced both mitochondria-mediated apoptosis and the autophagic cell death of HepG2 and MHCC97-L cells by suppressing the activity of Akt and up-regulating MAPK p38 signaling [32]. Likewise, Yokoyama et al. observed Vitamin K2 induced both autophagy and apoptosis simultaneously in HL-60 leukemia cells [33]. In our study, both apoptosis and autophagy were observed in Huh7 cells. However, the possibility that autophagy or apoptosis either alone or concomitantly contribute to 4-HPPP-induced cell death of Huh7 cells remains to be clarified.

Finally, we tested the protein expression of γH2AX to examine 4-HPPP-induced DNA damage of HCC cells. The results of γH2AX assessment demonstrated that 4-HPPP induces significant DNA damage in HCC cells, suggesting that 4-HPPP-induced DNA damage may contribute to the apoptosis and autophagy of HCC cells. These observations are similar to the results of Kim et al. who found that a certain phenoxyphenol analog, *N*1-hydroxy-*N*8-(4-phenoxyphenol)octanedianide (MHY218) significantly induced apoptosis and G2/M arrest through downregulation of NF-κB of human colon cancer HCT116 cells and gastric cancer AGS cells [18,34], reflecting a peculiarity of phenoxyphenol derivatives in anti-cancer treatment.

Interestingly, we noted that a significantly higher level of γH2AX in untreated Ha22T cells compared to untreated Huh7 cells. Generally, γH2AX was thought to be a stress marker, and a high level of γH2AX may indicate that cells are under stress, including oxidative stress [35] and replication stress [36]. However, the basal level of γH2AX may depend on cell type. For example, Huo’s study showed that a high level of γH2AX was observed in untreated benign Barrett’s epithelial cells [37], and this may explain the discrepancy in basal levels of H2AX between Huh7 and Ha22T cells. Therefore, the high basal γH2AX level seems to be the correlated 4-HPPP resistance of Ha22T cells.

Many studies have shown that tubulin inhibitors cause mitotic arrest [6,38], whereas some studies have shown that tubulin inhibitor-induced S-phase arrest could be observed. For example, Davis’s work reported that halogenated derivatives of acetamidobenzoyl ethyl ester target tubulin inhibiting cellular proliferation involved the S-phase arrest in acute lymphoblastic leukemia (CEM) [39]. More recently, 9-(4-vinylphenyl)noscapine (VinPhe-Nos), a novel analog of the noscapine alkaloid through the hyperacetylation of α-tubulin, is known to suppress the microtubule dynamics and finally cause S-phase arrest in breast cancer MDA-MB-231 cells [40]. Our preliminary results also showed that 4-HPPP did affect the distribution of cell cycle and cause an accumulation of S-phase dramatically at 48 h treatment (Table 1). Therefore, our further study will examine whether 4-HPPP affects the acetylation of tubulins and whether the down-regulation of tubulin expression could mainly contribute to the anti-proliferation and apoptosis in HCC cells.

Interestingly, between the two cell lines, the inhibitory effect of 4-HPPP was more pronounced in Huh7 cells than in HA22T cells, as shown by proliferation, apoptosis, autophagy and DNA damage detection assays. After treatment with 4-HPPP, the cell proliferation was markedly reduced in Huh7 cells and only weakly reduced in HA22T cells. Similarly, 4-HPPP significantly induced more apoptosis in Huh7 cells than in Ha22T cells. Meanwhile, elevated AVOs were observed in Huh7 cells, while no AVOs could be detected in Ha22T cells. The expression of autophagy-related LC3B was consistent with the increased formation of AVOs in Huh7 cells, but not in Ha22T cells, suggesting a resistance-causing effect of 4-HPPP on Ha22T cells. Finally, the γH2AX assessment results demonstrated that 4-HPPP induces significant DNA damage in Huh7 cells in a dose-dependent manner, while the dose-dependent effect was undetectable in Ha22T cells.

As per the results from above, Ha22T was considered relatively resistant to 4-HPPP compared to Huh7 and was thus used in the subsequent western blot assay to investigate the possible mechanism of anti-cancer effects of 4-HPPP in both HCC cells. The involvement of Akt, ERK signaling pathway and Survivin contribute to the apoptosis or resistance of HCC cell lines. Our results showed that 4-HPPP decreased the levels of survival-related proteins p-Akt (Ser^473^ and Thr^450^) and PCNA on Huh7 cells. On the contrary, 4-HPPP increased the levels of survival-related proteins p-Akt (Ser^473^ but not Thr^450^) and survivin on Ha22T cells. Because the ERK/MAPK pathway is associated with chemoresistance, it is being considered as a therapeutic target in the targeted molecular therapy of severe types of cancers. Zhang et al. showed that resistance to 5-fluorouracil (5-FU) was significantly associated with a basal phosphorylation level of ERK in HCC cell lines, and p-ERK was considered a potential biomarker predictive of sensitivity to chemo-drugs in treating HCC [41].

Feng et al. showed that ERK1 and ERK2 are over-expressed in HepG_2_-ADM (adriamycin-resistant HepG_2_) cell line, indicating these two kinases as potential targets for treating multi-drug resistant HCC cells [42]. Likewise, the alarmingly abundant level of p-ERK in Ha22T cells compared to Huh7 cells may imply the resistance of Ha22T to 4-HPPP treatment. Furthermore, the results of our study showed the ratio of viable cells were significantly decreased after treatment with 4-HPPP and PD98059, a selective inhibitor targeting mitogen-activated protein or extracellular signal-regulated kinase kinase 1 (MEK1), an upstream ERK kinase in Ha22T cells, suggesting that constitutive activation of ERK may contribute to the 4-HPPP resistance of Ha22T (Figure 9A), whereas pre-treatment with pan-Akt inhibitor did not affect the cell viability as compared with 4-HPPP alone (Figure 9B). Our study implicated that the blockade of ERK could synergistically enhance the inhibitory effect of 4-HPPP on the growth of Ha22T, suggesting that targeting ERK pathways may be a way to sensitize HCC cells such as Ha22T toward 4-HPPP treatment.

## 4. Materials and Methods

### 4.1. Source of 4-[4-(4-Hydroxyphenoxy)phenoxy]phenol 

4-[4-(4-Hydroxyphenoxy)phenoxy]phenol (4-HPPP) was purchased from the chemical supplier Enamine^TM^ (Kiev, Ukraine). 4-HPPP was dissolved in dimethyl sulfoxide (DMSO, less than 0.01%) at 10 mM as a stock solution.

### 4.2. Reagents

Medium DMEM, F-12, fetal bovine serum (FBS), the antibiotics penicillin G and streptomycin, and trypan blue were purchased from Gibco (Gaithersburg, MD, USA). DMSO and propidium iodide (PI) were purchased from Sigma-Aldrich (St. Louis, MO, USA). The antibodies against the following proteins survivin (#sc-17779), γH2AX (#sc-101696), phosphorylated Akt (Ser^473^; #sc-7985-R), Bid (#sc-11423) and PCNA (#sc-56) were purchased from Santa Cruz Biotechnology (Santa Cruz, CA, USA). LC3B (#2775) was purchased from Cell Signaling Technology, Inc. (Danvers, MA, USA) Phosphorylated ERK1/2 (Thr^202^/Tyr^204^, Thr^185^/Tyr^187^; #05-797R) was purchased from Merck Millipore (Billerica, MA, USA). Phosphorylated Akt (Thr^450^; #3188-1) was purchased from Epitomics (Burlingame, CA, USA). Akt1 (#ab32505) was purchased from Abcam (Cambridge, UK). PI3K (#GTX111173), Bax (#GTX109683), GAPDH (#GTX627408) and α-tubulin (#GTX102078) were purchased from GeneTex (Irvine, CA, USA). β-actin (#BD612656) was purchased from BD Biosciences (Franklin Lakes, NJ, USA). Caspase 3 (#IMG144A) was purchased from IMGENEX (San Diego, CA, USA). Horseradish peroxidase (HRP)-conjugated secondary antibodies (#20102 for goat anti-mouse IgG, #20202 for goat anti-rabbit IgG, Tainan, Taiwan) were purchased from Leadgene Biomedical Inc. Annexin V-FITC kit was purchased from Strong Biotech Co. (#AVK050, Taipei, Taiwan). Acridine orange (AO) was purchased from Sigma-Aldrich.

### 4.3. Cell Culture 

The poorly-differentiated human HCC cell line Ha22T was established from hepatoma [43] (BCRC #60168, Bioresources Collection and Research Center, Hsinchu, Taiwan) and Huh7 is a well differentiated hepatocyte derived cellular carcinoma cell line [44] (JCRB0403, JCRB, Cell Bank, Tokyo, Japan) was kindly gifted by Dr. JC Lee (Kaohsiung Medical University, Kaohsiung, Taiwan) [45], and the rat liver epithelial cell Clone 9 was kindly gifted by Dr. Ming-Hong Tai, Institute of Biomedical Sciences, National Sun-yat Sen University, Kaohsiung, Taiwan. All cell lines were incubated in a 100 mm^2^ petri-dish with 1:1 ratio of DMEM:F-12 medium and supplemented with 2 mM glutamine, 8% fetal bovine serum, and 100 units/mL penicillin and 100 μg/mL streptomycin (Gibco) in a 5% CO_2_ humidified atmosphere at 37 °C.

### 4.4. Cell Growth Assessment

The assessment of cell growth was performed according to the previous work with minor modifications [15]. In brief, a total of 5 × 10^4^ cells was seeded onto well in a 12-well plate and treated with phosphate-buffered saline (PBS) (Sigma-Aldrich) as different concentrations indicated for 48 and 72 h. After incubation, cell viability analysis was performed by trypan blue dye assay combined with an automated cell counter Countess™ according to the manufacturer’s instruction (Invitrogen, Carlsbad, CA, USA). The values of the half-maximum inhibitory concentration (IC_50_) for the inhibition study of activity was calculated using the software SigmaPlot^TM^ v12 (Systat Inc., Chicago, IL, USA).

### 4.5. Colony Formation Assay

To examine whether 4-HPPP affected the long-term proliferation of HCC and the rat liver Clone 9 cells, the colony formation assay was performed [46]. In brief, the density of seeding cell lines depended on their potential of cellular proliferation. Therefore, 50 cells of Clone 9, 400 cells of Ha22T and 1500 cells of Huh7 were seeded onto a 12-well plate 24 h respectively. The medium were then refreshed with indicated concentrations of 4-HPPP (from 0.5 to 10 μM) for 10 days (Clone 9) and 7 days (Ha22T and Huh7) respectively. Afterward, the colonies were fixed with 4% paraformaldehyde and stained with 0.1% *w*/*v* giemsa. The number of colonies was analyzed by Image-Pro (Media Cybernetics; Carlsbad, CA, USA).

### 4.6. Zebrafish Xenograft Assay

The zebrafish xenograft assay was used for validating the inhibitory effect of 4-HPPP on cell growth of Huh7 cells. The zebrafish assay complied with the 3R principles (Reduction, Replacement, and Refinement) and the approval protocol (IACUC Approval No. KMU-IACUC-105051) by Institutional Animal Care and Use Committee (IACUC) of Kaohsiung Medical University, Kaohsiung, Taiwan. Huh7 cells were labeled with the red fluorescent lipophilic cationic indocarbocyanine tracer 1,1′-dioctadecyl-3,3,3′,3′-tetra-methylendocarboxyamine (DiI) for tracking in the larvae prior to the zebrafish xenograft assay. The procedure was performed according to a previous study with minor modifications [47]. Briefly, the 2 days postfertilization (dpf) zebrafish embryos were anesthetized with 0.01% of tricaine and transplanted with about 200 human HCC cells per embryo. Cells then were resuspended in Hanks balanced salt solution and were injected into the yolk sac of the embryos. The embryos were then incubated in water with 1 μM of 4-HPPP for 24 and 48 h postinjection respectively. Afterward, the photographs were taken by an inverted phase-contrast microscope (Nikon Eclipse TE2000-U, Tokyo, Japan).

### 4.7. DAPI Staining

A total of 2 × 10^4^ Huh7 and Ha22T cells were seeded in 12-well plates and treated with 10 μM 4-HPPP for 72 h respectively. Cells were fixed in fixative (9:1 ratio of methanol:acetic acid) and washed with PBS. Cells were stained with DAPI for 5 min and washed twice with PBS. The nuclei were photographed by an epi-fluorescent microscope and were further analyzed by ImagePro software.

### 4.8. The Assessment Apoptosis Assay

The apoptosis of Ha22T and Huh7 cells was detected by flow cytometry-based annexin V-fluorescein isothiocyanate (FITC) staining kit purchased from Strong Biotech Co. (Taipei, Taiwan) [46]. Apoptotic cells were to be detected by the binding of annexin V-FITC with phosphatidylserine (PS) on the outer membrane, which is a hallmark of apoptosis. Briefly, cells were incubated with indicated concentrations of 4-HPPP (from 0.5 to 10 μM) for 72 h respectively. The cells were harvested and then stained with 10 μg/mL of annexin V-FITC and 20 μg/mL of PI, then cells were analyzed using an LSR II Flow Cytometer (Becton, Dickinson and Company Biosciences, San Jose, CA, USA).

### 4.9. Acridine Orange (AO) Staining

The autophagy of Ha22T and Huh7 cells was detected by flow cytometry-based acridine orange (AO) dye [48]. 1 × 10^5^ cells was seeded and treated with indicated concentrations (from 0.5 to 10 μM) of 4-HPPP for 24 and 48 h respectively. The cells were then harvested and stained with 1 μg/mL AO at 25 °C for 15 min. Cells were washed with PBS and analyzed suing LSR II flow cytometer (Excitation: 488-nm and Emission: 515-nm for green fluorescence and 650-nm for red fluorescence respectively).

### 4.10. Western Blotting Assay

The 4-HPPP-treated cells were lysed using RIPA buffer (50 mM Tris-HCl, pH 7.5, 0.15 M NaCl, 1% NP-40, 0.5% Sodium deoxycholate and 0.1% SDS). 20 μg of protein were resolved using a sodium dodecyl sulfate-polyacrylamide gel electrophoresis (SDS-PAGE) and transferred to a polyvinylidene fluoride (PVDF) membrane. The membrane was blocked with Tris-based saline-0.5% Tween-20 buffer (50 mM Tris-HCl pH 7.4, 150 mM NaCl, 0.1% Tween-20) containing 5% skim milk at 4 °C, and incubated with primary antibodies and the horseradish peroxidase-conjugated secondary antibody sequentially. The signals of specific proteins were detected using a chemiluminescence kit (Advansta Corp., Menlo Park, CA, USA) according to the manufacturer’s manual [46].

### 4.11. Assessment of ERK and Akt Inhibition

To further determine the role of ERK and Akt in 4-HPPP-induced anti-HCC effect, a total of 5 × 10^4^ Ha22T cells were seeded into a 12-well plate. The used concentration of MEK1/ERK inhibitor PD98059 was according to Wang’s work with minor modification [49]. Cells then were pre-treated for 6 h with MEK1/ERK PD98059 (EI-360-0005; Enzo Life Sciences Inc., Farmingdale, NY, USA) and Akt-specific inhibitor and MK-2206 (s1078, Selleck Chemicals, Houston, TX, USA) before 4-HPPP. Afterward, the viable cells were counted using the trypan blue exclusion assay respectively.

### 4.12. Cell Cycle Distribution

The distributions of cell cycle were assessed using a flow cytometer-based PI staining described previously [50]. In brief, cells were treated with indicated concentrations of 4-HPPP for 24 and 48 h respectively. Harvested cells were 70% ethanol-fixed overnight. After centrifugation, the cell pellets were stained with 10 mg/mL PI and 10 mg/mL RNase A in darkness. The cells were analyzed using FlowJo software (Treestar, Inc., San Carlos, CA, USA).

### 4.13. Statistical Analysis 

All experiments were conducted in triplicate. Data are presented as means ± SD (standard deviation). The differences were statically analyzed by one-way analysis of variance (ANOVA) using SigmaPlot software except for analyzing the results of the colony formation assay. * *p* < 0.05 (vehicle vs. 4-HPPP) was considered significant statistically.

## 5. Conclusions

**Figure 10:**
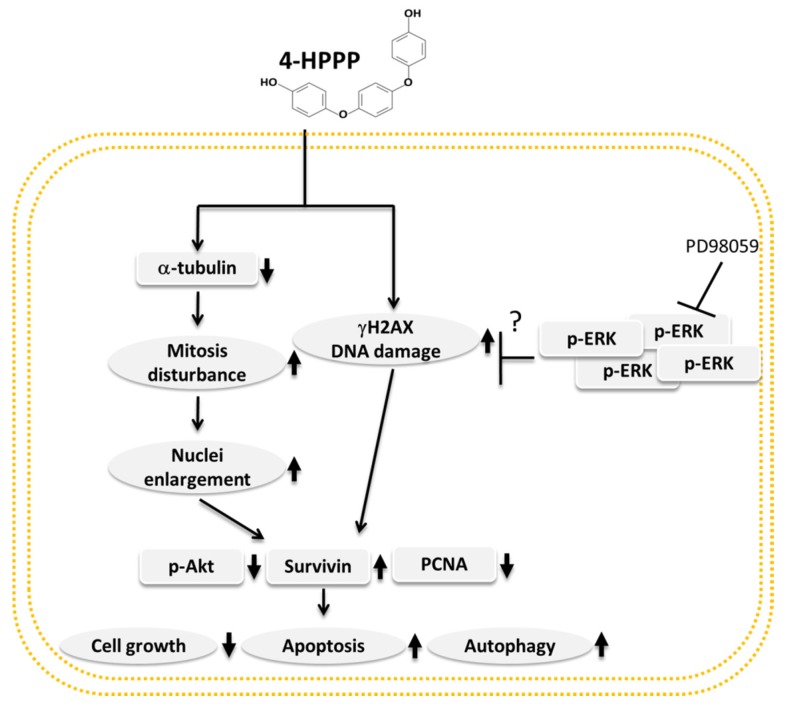
Proposed mechanism of 4-HPPP-induced apoptosis, autophagy and reduced expression of α-tubulin in HCC cells.

The 4-phenoxyphenol derivative 4-HPPP exerts its anti-HCC activity including anti-proliferation, induction of apoptosis through downregulating the ERK pathways, and anti-proliferative effect in human HCC cells, as demonstrated. Our results showed that 4-HPPP induced anti-growth and the induction of apoptosis in HCC cells accompanied with autophagy. Besides, the down-regulated expression of α-tubulin may contribute to the 4-HPPP-induced apoptosis of HCC cells, suggesting the chemotherapeutic or chemopreventive potential for HCC treatment in future (Figure 10). The results of our study demonstrated the discrepant anti-HCC effect of phenoxyphenol derivative 4-HPPP, and the underlying mechanism would be worthy for further elucidation.

## Figures and Tables

**Table 1:** The distributions of cell cycle in 4-HPPP-treated Huh7 cells.

	% Phase	24 h		48 h		4-HPPP (μM)
		0	10	0	10	
Huh7	sub-G_1_	1.3 ± 0.2	4.4 ± 0.6	4.0 ± 0.6	13.1 ±6.9	
	G_1_	58.1 ± 1.8	74.9 ± 1.5 *	50.9 ± 0.1	16.4 ± 0.2 *	
	S	14.0 ± 0.4	15.0 ± 0.2	12.4 ± 0.3	40.1 ± 3.6 *	
	G_2_/M	24.5 ± 1.6	6.9 ± 0.9 *	19.5 ± 0.6	17.7 ± 1.6	

* *p* < 0.001.

## Abbreviations

Akt	Protein kinase B
AO	Acridine orange
AVOs	Acidic vesicular organelles
Ctrl	Control
COX-2	Cyclooxygenase-2
CQ	Chloroquine
DAPI	4′,6-Diamidino-2-phenylindole
DiI	1,1′-Dioctadecyl-3,3,3′,3′-Tetramethylindocarbocyanine Perchlorate
DMSO	Dimethyl sulfoxide
ERK	Extracellular signal-regulated kinase
GAPDH	Glyceraldehyde-3-phosphate dehydrogenase
γH2AX	Gamma-H2A histone family, member X
HCC	Hepatocellular carcinoma
4-HPPP	4-[4-(4-Hydroxyphenoxy)phenoxy]phenol
IC_50_	Half-maximum inhibitory concentration
LC3B	Microtubule-associated protein-1 light chain-3B
MEK1	Mitogen-activated protein or extracellular signal-regulated kinase kinase 1
MMP	Matrix metalloproteinases
MTT	3-(4,5-Dimethylthiazol-2-yl)-2,5-diphenyltetrazolium bromide
PCNA	Proliferating cell nuclear antigen
PI	Propidium iodide
PS	Phosphatidylserine
SD	Standard deviation
SDS-PAGE	Sodium dodecyl sulfate-polyacrylamide gel electrophoresis
